# Vimentin is a potential prognostic factor for tongue squamous cell carcinoma among five epithelial–mesenchymal transition-related proteins

**DOI:** 10.1371/journal.pone.0178581

**Published:** 2017-06-01

**Authors:** Pei-Feng Liu, Bor-Hwang Kang, Yi-Min Wu, Ju-Hsin Sun, Liang-Ming Yen, Ting-Ying Fu, Yun-Chung Lin, Huei-Han Liou, Yaoh-Shiang Lin, Huei-Cin Sie, I-Chien Hsieh, Yu-Kai Tseng, Chih-Wen Shu, Yao-Dung Hsieh, Luo-Ping Ger

**Affiliations:** 1 Department of Medical Education and Research, Kaohsiung Veterans General Hospital, Kaohsiung, Taiwan; 2 Department of Biotechnology, Fooyin University, Kaohsiung, Taiwan; 3 Department of Otorhinolaryngology-Head and Neck Surgery, Kaohsiung Veterans General Hospital, Kaohsiung, Taiwan; 4 Graduate Institute of Aerospace and Undersea Medicine, National Defense Medical Center, Taipei, Taiwan; 5 Graduate Institute of Oral Health Sciences, College of Dental Medicine, Kaohsiung Medical University, Kaohsiung, Taiwan; 6 Division of Periodontics, Department of Dentistry, Kaohsiung Medical University Hospital, Kaohsiung, Taiwan; 7 Department of Medical Management, Kaohsiung Veterans General Hospital, Kaohsiung, Taiwan; 8 Department of Pathology and Laboratory Medicine, Kaohsiung Veterans General Hospital, Kaohsiung, Taiwan; 9 Department of Pathology, China Medical University Hospital, Taichung, Taiwan; 10 Department of Otolaryngology-Head and Neck Surgery, National Defense Medical Center, Taipei, Taiwan; 11 Department of Orthopedics, National Cheng Kung University Hospital, Tainan, Taiwan; 12 Department of Orthopedics, Show Chwan Memorial Hospital, Changhua, Taiwan; 13 Department of Stomatology, Kaohsiung Veterans General Hospital, Kaohsiung, Taiwan; 14 Department of Dentistry, Kaohsiung Veterans Gegeral Hospital, Pingtung Branch, Pingtung, Taiwan; 15 Institute of Biomedical Sciences, National Sun Yat-Sen University, Kaohsiung, Taiwan; University of North Carolina at Chapel Hill School of Medicine, UNITED STATES

## Abstract

We aimed to investigate the association of the expression levels of five epithelial–mesenchymal transition (EMT)-related proteins (Snail, Twist, E-cadherin, N-cadherin, and Vimentin) with tumorigenesis, pathologic parameters and prognosis in tongue squamous cell carcinoma (TSCC) patients by immunohistochemistry of tissue microarray. The expression levels of Snail, E-cadherin, N-cadherin and Vimentin were significantly different between the tumor adjacent normal and tumor tissues. In tumor tissues, lower E-cadherin and higher N-cadherin levels were associated with a higher grade of cell differentiation, advanced stage of disease, and lymph node metastasis. However, higher Vimentin expression was associated with poor cell differentiation and lymph node metastasis. Patients with low E-cadherin expression had poor disease-specific survival (DSS). Conversely, positive N-cadherin and higher Vimentin expression levels were associated with poor DSS and disease-free survival. Notably, our multivariate Cox regression model indicated that high Vimentin expression was an adverse prognostic factor for DSS in TSCC patients, even after the adjustment for cell differentiation, pathological stage, and expression levels of Snail, Twist, E-cadherin, and N-cadherin. Snail, E-cadherin, N-cadherin, and Vimentin were associated with tumorigenesis and pathological outcomes. Among the five EMT-related proteins, Vimentin was a potential prognostic factor for TSCC patients.

## Introduction

Tongue squamous cell carcinoma (TSCC) is one of the most common cancers of the oral cavity. It is more aggressive than other forms of oral cancer, with a propensity for rapid local invasion and spread [1] along with a high recurrence rate [2]. Because the tongue is rich in lymphatic vessels and neurovascular bundles, the incidence of neck nodal metastasis is higher [3]. Despite overall improvements in surgical and medical management, the clinical outcomes of TSCC patients have remained unchanged, indicating the immediate need for new prognostic biomarkers for TSCC patients.

Epithelial—mesenchymal transition (EMT), recently identified as a key process in carcinogenesis, invasion, and metastasis, is a predictor of TSCC progression [4]. It plays a critical role in promoting metastasis in epithelial carcinoma, accompanied by a decrease in the expression of epithelial markers, including cell-surface E-cadherin, and an increase in the expression of mesenchymal markers, such as transcription factors Snail and Twist, and cell-surface N-cadherin, as well as cytoskeletal Vimentin [5,6]. E-cadherin, a calcium-dependent transmembrane glycoprotein, is expressed in most epithelial cells for cell polarity and tissue structure [7]. Snail and Twist are transcription factors that repress E-cadherin expression in epithelial tumors to promote metastasis by disassembling cellular adhesion junctions [8]. N-cadherin induces a mesenchymal-scattered phenotype associated with reduced E-cadherin levels in squamous cell carcinoma [9]. Vimentin is an intermediate filament protein that is ubiquitously expressed in normal mesenchymal cells to maintain the cellular architecture and tissue integrity, and it also participates in tumorigenesis, EMT, and the metastatic spread of cancer [10].

Many studies have used immunohistochemistry to investigate the expression levels of EMT-related biomarkers in TSCC patients. However, the tumorigenic and prognostic significance of these biomarkers was studied in a relatively small cohort with short follow-up [11–14]. Moreover, combinatorial assessment of Snail, Twist, E-cadherin, N-cadherin, and Vimentin expression levels in tumorigenesis and prognosis of TSCC has not been reported previously. In this study, the expression levels of these five EMT-related markers and their correlations with tumorigenesis, pathological outcomes, and survival were extensively investigated in 248 TSCC patients.

## Materials and methods

### Patients and tissue subjects

Specimens of TSCC tissues (n = 248) and corresponding tumor adjacent normal (TAN, n = 235) tissues were obtained from the Department of Pathology, Kaohsiung Veterans General Hospital between 1993 and 2006. The 2002 American Joint Committee on Cancer (AJCC) system was used to classify pathological TNM stages at the time of initial resection of the tumor. Informed consent forms were provided by all patients involved in this study, and the protocol of this study was approved by the Institutional Review Board at Kaohsiung Veterans General Hospital (IRB number: VGHKS11-CT12-13).

### Tissue microarray construction

The detailed procedures of tissue microarray (TMA) construction were described in our previous study [15]. The TMA block consisted of 129 cores, including 43 trios with each trio containing 2 cores from the tumor tissue and 1 core from the non-cancerous epithelium of the same patient. After exclusion of incorrectly identified tissues, cores with too few normal (or tumor) cells (<20 cells), missing cores and those of poor quality, only 485 tumor cores and 212 TAN cores were stained or scored in this study. After construction, 4-μm-thick sections of the TMA blocks were cut and processed for immunohistochemistry.

### Immunohistochemistry (IHC)

IHC of all proteins was performed using the Novolink Max Polymer Detection System (Leica, Newcastle Upon Tyne, United Kingdom). The sections were deparaffinized in xylene, rehydrated in graded ethanol, and washed for 5 min with phosphate-buffered saline (PBS). The sections were boiled and immersed in Tris-EDTA (10 mM, pH 9.0) for 10 min at 125°C for antigen retrieval. Endogenous peroxidase activity was blocked for 30 minutes with 3% hydrogen peroxide in methanol. The sections were incubated with primary antibodies [anti-Snail, Abcam (ab53519), 1:100; anti-Twist, Santa Cruz (sc-15393), 1:50; anti-E-cad, BD Biosciences (610181), 1:200; anti-N-cad, Leica (NCL-L-N-Cad), 1:200; anti-Vimentin, SPRING (M3200), 1:400] overnight at 4°C in a humidified chamber after blocking. The sections were incubated with horseradish peroxidase-labeled secondary antibody for 10 minutes at room temperature after washing in PBS and counterstained with hematoxylin [16].

### IHC analysis and scoring

**Fig 1:**
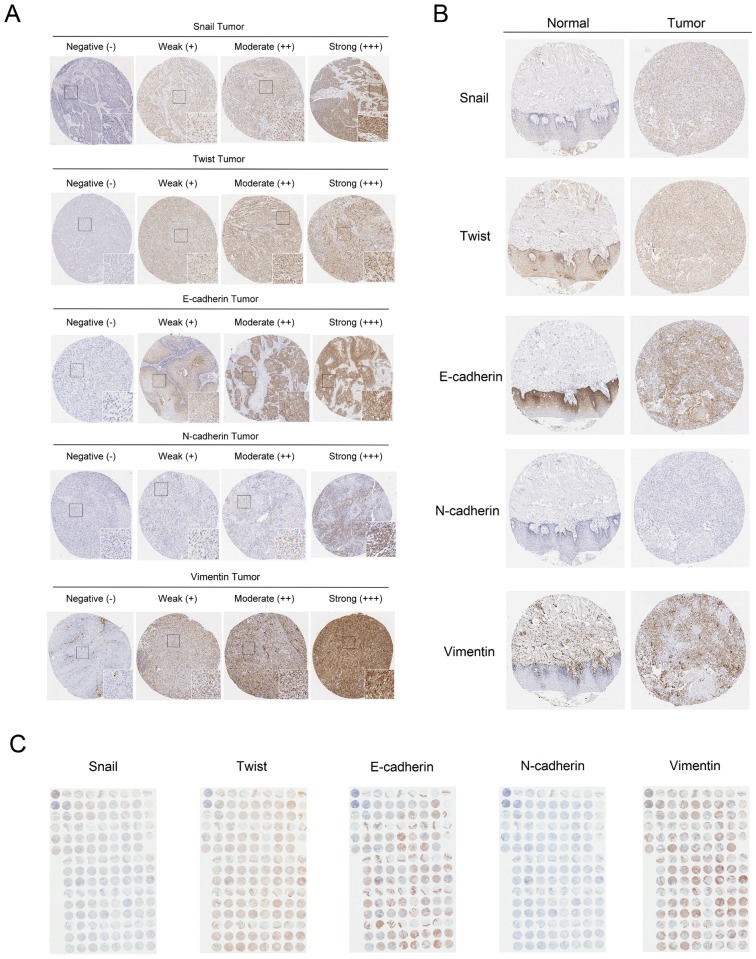
Immunoreactivity of Snail, Twist, E-cadherin, N-cadherin and Vimentin in TSCC. (A) The representative photomicrographs for negative (–), weak (+), moderate (++) and strong (+++) staining of Snail, Twist, E-cadherin, N-cadherin and Vimentin in TSCC tissues. (B) Comparative representative photomicrographs of Snail, Twist, E-cadherin, N-cadherin and Vimentin between normal and TSCC tissues. (C) The representative photomicrographs of TMA sections for Snail, Twist, E-cadherin, N-cadherin and Vimentin staining.

The scoring of IHC staining was performed by a senior technician under the supervision of an oral cancer pathologist as described in our previous study [15]. As a first step, digital images of the IHC-stained TMA slides were evaluated by an oral cancer pathologist (Dr. Ting-Ying Fu) along with a senior technician (Mr. Ju-Hsin Sun) until all discrepancies were resolved. Subsequently, the technician independently reviewed all slides, except for cores with incorrect and uncertain content, which need to be scored by the pathologist. Afterward, the pathologist randomly re-evaluated 15% of the core samples. If the agreement of IHC scores between the pathologist and technician was < 95%, the pathologist evaluated slides again with the technician until all discrepancies were resolved. After that, the senior technician re-scored all slides until the scoring agreement was ≥ 95% among random core samples (5–20%) evaluated by the pathologist. Approximately 45% of the cores were scored (for those that were incorrect, special, or uncertain) or re-evaluated (for random core samples) by the pathologist. In the final re-evaluation of the IHC scores, the agreement between the pathologist and technician for Snail, Twist, E-cadherin, N-cadherin, Vimentin expression was 96%, 96%, 97%%, 98%, and 95%, respectively. A semi-quantitative approach was used to grade the immunoreactivity. The intensity score of nuclear staining (for Snail and Twist), cell membranous staining (for E-cadherin and N-cadherin), and cytoplasmic staining (Vimentin) was measured using a numerical scale (-, negative expression; +, weak expression; ++, moderate expression; and +++, strong expression; Fig 1A). The percentage of cells stained at each intensity level was graded as 0 (<5%), 1 (5–25%), 2 (26–50%), 3 (51–75%), and 4 (>75%). The intensity score and percentage of positive cells were multiplied to derive the final scores. For survival analysis, expression levels of all proteins were dichotomized into low expression and high expression with the cutoff value based on a receiver operating characteristic (ROC) curve analysis. The cutoff values were 4, 5, 3, 0, and 2 for Snail, Twist, E-cadherin, N-cadherin, and Vimentin, respectively. Moreover, the cutoff value of 0 was defined as negative expression, and the cutoff value of >0 was defined as positive expression for N-cadherin.

### Statistical analysis

The Wilcoxon matched pairs signed rank test was used to evaluate the differential expression of Snail, Twist, E-cadherin, N-cadherin, and Vimentin between paired tissues (TSCC vs. paired TAN). The correlation between the expression score of each protein and pathologic parameters was evaluated with Kruskal-Wallis one-way ANOVA, one-way ANOVA, Mann-Whitney U test, or Student’s t-test. Disease-specific survival (DSS) was measured from the time of initial resection of the primary tumor to the date of cancer-specific death or the date of last follow-up (October 2010). Disease-free survival (DFS) was calculated from the date of initial resection of the primary tumor to the date of recurrence or that of last follow-up. The Kaplan-Meier method was used to estimate cumulative survival curves. The log-rank test was used to compare the different survival curves. The independent predictors of survival were determined by multivariate Cox proportional hazards modeling. P values < 0.05 were defined as statistically significant. The raw clinical data along with expression scores of five proteins are provided in the supporting information (S1 Table).

## Results

### Association of the expression levels of five EMT-related biomarkers with tumorigenesis

The levels of Snail, Twist, E-cadherin, N-cadherin, and Vimentin were analyzed in TSCC tissue cores and were compared with the levels of the respective proteins in paired TAN tissue cores (Fig 1B) on the TMA (Fig 1C). The results showed that higher expression levels of Snail (187 cases, p<0.001) and Vimentin (206 cases, p<0.001) and lower expression levels of E-cadherin (208 cases, p<0.001) and N-cadherin (210 cases, p<0.001) were found in TSCC tissues compared to the paired TAN tissues (Table 1).

**Table 1:** The pairwise comparisons of Snail, Twist, E-cadherin, N-cadherin, and Vimentin expression between TSCC and corresponding tumor adjacent normal tissues.

Variables	No.	Tumor adjacent normal		Tumor		Z	*p*-value*
		mean±SD	Median	mean±SD	Median		
Snail	187	3.47±1.30	3.00	4.03±1.56	4.00	4.728	**<0.001**
Twist	195	4.77±1.35	5.00	4.84±1.65	5.00	1.010	0.312
E-cadherin	208	5.28±1.10	5.00	3.80±1.38	4.00	-9.860	**<0.001**
N-cadherin	210	0.88±1.61	0.00	0.20±0.73	0.00	-5.498	**<0.001**
Vimentin	206	0.12±0.52	0.00	1.62±1.43	2.00	9.998	**<0.001**

Abbreviations: TSCC, tongue squamous cell carcinoma; SD, standard deviation.

*p-values were estimated by Wilcoxon signed-rank test.

### Association of expression levels of the five EMT-related biomarkers with demographic and pathological outcomes of TSCC patients

The association of expression levels of EMT markers with various demographic and pathological outcomes was examined in TSCC tissues (Table 2). Snail expression was significantly increased in advanced stage disease (p = 0.037). Moreover, N-cadherin expression was significantly increased in samples connected with a higher grade of cell differentiation (p<0.001), advanced stage of disease (p = 0.023), and lymph node metastasis (p = 0.007). Similarly, Vimentin expression was significantly increased in samples connected with a higher grade of cell differentiation (p = 0.006) and with lymph node metastasis (p = 0.039). In contrast, decreased expression of E-cadherin was found in samples connected with a higher grade of cell differentiation (p<0.001), advanced stage of disease (p = 0.013), and with lymph node metastasis (p = 0.007).

**Table 2:** The comparisons of Snail, Twist, E-cadherin, N-cadherin, and Vimentin expression with various demographic and pathologic outcomes in TSCC patients.

Variable	No. (%)	Snail		Twist		E-cadherin		N-cadherin		Vimentin	
		Mean±SD	*p value*	Mean±SD (median)	*p value*	Mean±SD (median)	*p value*	Mean±SD (median)	*p value*	Mean±SD (median)	*p value*
Sex											
Female	30 (12.1)	3.87±1.61 (4.00)	0.620*	4.67±1.54 (5.00)	0.573*	3.83±1.42 (4.00)	0.896*	0.20±0.66 (0.00)	0.874*	1.17±1.44 (0.50)	0.054*
Male	218 (87.9)	4.02±1.56 (4.00)		4.85±1.67 (5.00)		3.80±1.38 (4.00)		0.18±0.69 (0.00)		1.70±1.41 (2.00)	
Age, y											
≦40	48 (19.4)	4.00±1.47 (4.00)	0.435^†^	5.13±1.66 (6.00)	0.206^‡^	3.81±1.41 (4.00)	0.894^†^	0.38±1.04 (0.00)	0.561^‡^	1.54±1.38 (2.00)	0.849^†^
41–50	80 (32.3)	4.05±1.65 (4.00)		4.73±1.84 (5.00)		3.74±1.51 (4.00)		0.15±0.58 (0.00)		1.58±1.57 (1.50)	
51–60	67 (27.0)	3.76±1.71 (4.00)		4.61±1.71 (5.00)		3.78±1.31 (4.00)		0.15±0.61 (0.00)		1.75±1.35 (2.00)	
>60	53 (21.4)	4.23±1.31 (4.00)		4.98±1.22 (5.00)		3.92±1.27 (4.00)		0.09±0.45 (0.00)		1.68±1.34 (2.00)	
Cell differentiation											
Well	26 (10.5)	3.88±1.34 (4.00)	0.512^†^	4.85±1.64 (5.00)	0.558^‡^	4.42±1.06^a^^b^ (5.00)	**<0.001**^‡^	0.04±0.20^d^ (0.00)	**<0.001**^‡^	1.19±1.06^f^ (1.00)	**0.006**^‡^
Moderate	205 (82.7)	3.98±1.57 (4.00)		4.88±1.60 (5.00)		3.86±1.28^a^^c^ (4.00)		0.13±0.54^e^ (0.00)		1.59±1.36^g^ (2.00)	
Poor	17 (6.9)	4.41±1.87 (5.00)		4.18±2.21 (5.00)		2.18±1.85^b^^c^ (2.00)		1.06±1.60^d^^e^ (0.00)		2.88±2.00^f^^g^ (3.00)	
AJCC pathological stage											
I, II	168 (67.7)	3.86±1.55 (4.00)	0.037*	4.80±1.59 (5.00)	0.690*	3.95±1.35 (4.00)	0.013*	0.12±0.56 (0.00)	**0.023**^§^	1.57±1.35 (2.00)	0.294*
III, IV	80 (32.3)	4.30±1.56 (5.00)		4.89±1.79 (5.00)		3.49±1.40 (4.00)		0.31±0.88 (0.00)		1.78±1.57 (2.00)	
T classification											
T1, T2	195 (78.6)	3.94±1.57 (4.00)	0.236*	4.81±1.62 (5.00)	0.696*	3.84±1.41 (4.00)	0.400*	0.16±0.63 (0.00)	0.443*	1.68±1.45 (2.00)	0.342*
T3, T4	53 (21.4)	4.23±1.55 (5.00)		4.91±1.78 (5.00)		3.66±1.27 (4.00)		0.25±0.85 (0.00)		1.47±1.31 (2.00)	
N classification											
N0	196 (79.0)	3.96±1.54 (4.00)	0.427*	4.82±1.60 (5.00)	0.924*	3.92±1.34 (4.00)	0.007*	0.13±0.60 (0.00)	**0.007**^§^	1.54±1.33 (2.00)	0.039*
N1, N2	52 (21.0)	4.15±1.67 (4.00)		4.85±1.87 (5.00)		3.35±1.47 (3.00)		0.37±0.91 (0.00)		2.00±1.70 (2.00)	

Abbreviations: TSCC, tongue squamous cell carcinoma; AJCC, American Joint Committee on Cancer.

*p values were estimated by Student’s t-test.

^†^ p values were estimated by one-way ANOVA test.

^‡^p values were estimated by Kruskal-Wallis one-way ANOVA test.

^§^p values were estimated by Mann-Whitney U test.

^a^p = 0.026;

^b^p<0.001;

^c^p<0.001;

^d^p = 0.005;

^e^p<0.001;

^f^p = 0.003;

^g^p = 0.005.

### Association of expression levels of the five EMT-related biomarkers with survival in TSCC patients

**Fig 2:**
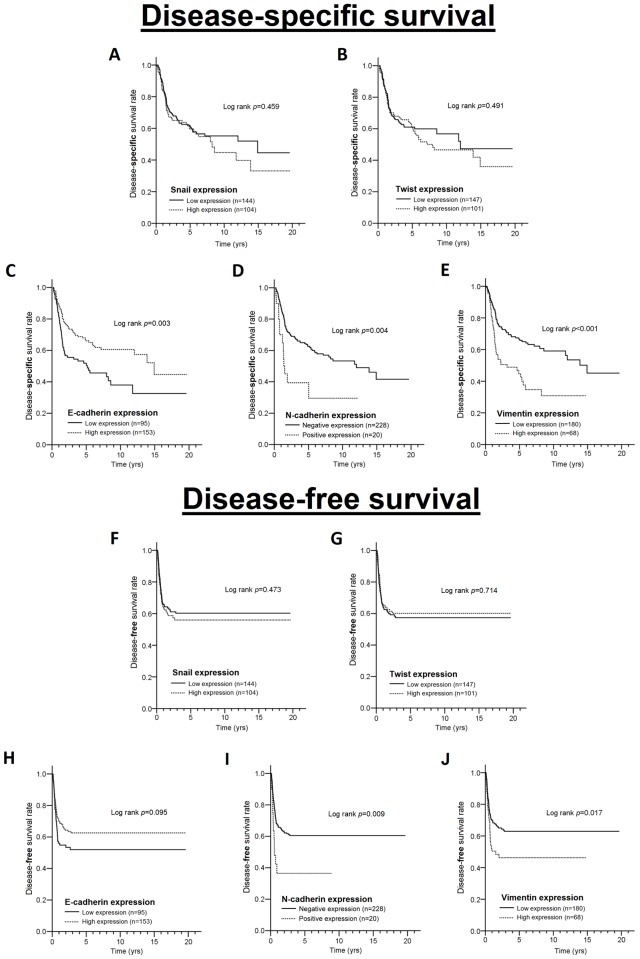
Kaplan-Meier survival curves for disease-specific survival according to (A) Snail expression status, (B) Twist expression status, (C) E-cadherin expression status, (D) N-cadherin expression status, and (E) Vimentin expression status in TSCC patients. Kaplan-Meier curves for disease-free survival according to (F) Snail expression status, (G) Twist expression status, (H) E-cadherin expression status, and (I) N-cadherin expression status.

In univariate analysis, the expression of Snail (Fig 2A and 2F) and Twist (Fig 2B and 2G) was not associated with DSS and DFS (Table 3). However, a low level of E-cadherin expression was correlated with worse DSS (p = 0.003, Fig 2C) but not associated with DFS (p = 0.095, Fig 2H). Moreover, patients with positive N-cadherin expression had poor DSS (p = 0.004, Fig 2D) and DFS (p = 0.009, Fig 2I). Patients with high Vimentin expression levels had poor DSS (p<0.001, Fig 2E) and DFS (p = 0.017, Fig 2J). After adjustment for cell differentiation and pathologic stage in the multivariate Cox regression model, high E-cadherin expression level was significantly correlated with longer DSS (adjusted hazard ratio [AHR] = 0.66; 95% CI = 0.45–0.97; p = 0.033; Table 3) but not with DFS (p = 0.20). Positive N-cadherin expression was still correlated with both shorter DSS (AHR = 1.88; 95% CI = 1.05–3.39; p = 0.034; Table 3) and DFS (AHR = 2.06; 95% CI = 1.11–3.81; p = 0.022; Table 3). Likewise, high Vimentin expression level was correlated with both shorter DSS (AHR = 2.05; 95% CI = 1.38–3.04; p<0.001; Table 3) and shorter DFS (AHR = 1.54; 95% CI = 1.01–2.34; p = 0.045; Table 3). These results confirmed that a high level of E-cadherin expression is an independent favorable prognostic factor for DSS, whereas positive N-cadherin and high Vimentin expression are independent adverse prognostic factors for both DSS and DFS in TSCC patients.

**Table 3:** The expression levels of Snail, Twist, E-cadherin, N-cadherin, and Vimentin for disease-specific survival and disease-free survival of TSCC patients.

Variable		No. (%)	Disease-specific survival		Disease-free survival	
			AHR (95% CI)	p value*	AHR (95% CI)	p value*
Snail expression	Low (0–4)	144 (58.1)	1.00		1.00	
	High (5–7)	104 (41.9)	1.00 (0.68–1.45)	0.979	1.11 (0.74–1.66)	0.624
Twist expression	Low (0–5)	147 (59.3)	1.00		1.00	
	High (6–7)	101 (40.7)	0.99 (0.67–1.45)	0.941	0.93 (0.62–1.40)	0.732
E-cadherin expression	Low (0–3)	95 (38.3)	1.00		1.00	
	High (4–7)	153 (61.7)	**0.66 (0.45–0.97)**	**0.033**	0.77 (0.51–1.15)	0.200
N-cadherin expression	Negative (0)	228 (91.9)	1.00		1.00	
	Positive (1–7)	20 (8.1)	**1.88 (1.05–3.39)**	**0.034**	**2.06 (1.11–3.81)**	**0.022**
Vimentin	Low (0–2)	180 (72.6)	1.00		1.00	
	High (3–7)	68 (27.4)	**2.05 (1.38–3.04)**	**<0.001**	**1.54 (1.01–2.34)**	**0.045**

Abbreviations: TSCC, tongue squamous cell carcinoma; AHR, adjusted hazard ratio; CI, confidence interval.

*p-value were adjusted for cell differentiation(moderate+poor vs. well) and AJCC pathological stage (stage III+IV vs stage I+II) by multiple Cox‘s regression.

The impact of E-cadherin on DSS and DFS was further analyzed by stratification based on demographic and pathological outcomes of patients, which has not been studied previously. Younger (≤50 yrs) patients with high level of E-cadherin expression had a longer DSS (AHR = 0.50; 95% CI = 0.30–0.83; p = 0.007, S2 Table). Moreover, a high level of E-cadherin was associated with better DSS in patients with moderate or poor cell differentiation (AHR = 0.66; 95% CI = 0.45–0.98; p = 0.038, S2 Table) and advanced stage of disease (III-IV, AHR = 0.57; 95% CI = 0.33–1.00; p = 0.048, S2 Table) as well as in patients who never received post-operative radiotherapy (AHR = 0.55; 95% CI = 0.34–0.89; p = 0.014, S2 Table). However, the expression level of E-cadherin was not associated with DFS, even after stratification analysis (S3 Table). Moreover, a high level of Vimentin was associated with poor DSS in younger patients (≤50 yrs; AHR = 2.53; 95% CI = 1.51–4.24; p<0.002, S4 Table), in patients with moderate or poor cell differentiation (AHR = 1.99; 95% CI = 1.34–2.97; p = 0.001, S4 Table), and in patients with advanced stage of disease (III-IV, AHR = 1.90; 95% CI = 1.07–3.38; p = 0.03, S4 Table) as well as in those without lymph node metastasis (N0; AHR = 1.97; 95% CI = 1.20–3.23; p = 0.008, S4 Table) but in those with smaller tumor size (T1-T2; AHR = 2.12; 95% CI = 1.34–3.37; p = 0.001, S4 Table). Additionally, high Vimentin expression was associated with poor DFS in patients with early pathological stage disease (I-II; AHR = 1.86; 95% CI = 1.13–3.08; p = 0.016, S5 Table) and in patients without lymph node metastasis (N0; AHR = 1.79; 95% CI = 1.11–2.88; p = 0.017, S5 Table). Nevertheless, the stratification analysis was not performed for N-cadherin expression due to the limited number of patients (n = 20) with positive expression.

### Vimentin as a potential prognostic factor among the five EMT-related proteins in TSCC patients

To investigate the interaction of expression of Snail, Twist, E-cadherin, N-cadherin, and Vimentin as well as pathologic outcomes with survival, the data on the expression levels of these proteins and outcome (cell differentiation and pathologic stage) were put into a Cox regression model. The results showed that Vimentin was still a less favorable prognostic factor for DSS in TSCC patients (AHR = 1.90; 95% CI = 1.26–2.87; p = 0.002; Table 4) after adjustment for cell differentiation, pathological stage, and expression of Snail, Twist, E-cadherin, and N-cadherin.

**Table 4:** Multivariate Cox’s regression analysis of Snail, Twist, E-cadherin, N-cadherin, and Vimentin for disease-specific survival and disease-free survival of TSCC patients.

Variable		No. (%)	Disease-specific survival		Disease-free survival	
			AHR (95% CI)	*p value*	AHR (95% CI)	*p value*
Snail expression	Low (0–4)	144 (58.1)	1.00		1.00	
	High (5–7)	104 (41.9)	0.85 (0.57–1.26)	0.419	0.99 (0.65–1.51)	0.954
Twist expression	Low (0–5)	147 (59.3)	1.00		1.00	
	High (6–7)	101 (40.7)	0.99 (0.68–1.46)	0.967	0.94 (0.62–1.41)	0.756
E-cadherin expression	Low (0–3)	95 (38.3)	1.00		1.00	
	High (4–7)	153 (61.7)	0.75 (0.50–1.11)	0.144	0.85 (0.56–1.30)	0.464
N-cadherin expression	Negative (0)	228 (91.9)	1.00		1.00	
	Positive (1–7)	20 (8.1)	1.57 (0.87–2.86)	0.135	1.82 (0.96–3.45)	0.066
Vimentin	Low (0–2)	180 (72.6)	1.00		1.00	
	High (3–7)	68 (27.4)	**1.90 (1.26–2.87)**	**0.002**	1.38 (0.88–2.16)	0.159
Cell differentiation	Well	26 (10.5)	1.00		1.00	
	Moderate, poor	222 (89.5)	**2.70 (1.08–6.73)**	**0.033**	**3.13 (1.15–8.57)**	**0.026**
AJCC pathological stage	I, II	168 (67.7)	1.00		1.00	
	III, IV	80 (32.3)	**2.98 (2.01–4.42)**	**<0.001**	1.03 (0.66–1.61)	0.882

Abbreviations: TSCC, tongue squamous cell carcinoma; AHR, adjusted hazard ratio; CI, confidence interval.

## Discussion

EMT plays important roles in cancer invasion and metastasis, involving Vimentin as a cytoskeletal regulator of migration of mesenchymal cells and cell-surface E/N-cadherin switch (the expression of E-cadherin is switched off and that of N-cadherin is switched on) mediated by key transcription factors such as Snail and Twist [8]. In this study, we found that Snail, E-cadherin, N-cadherin, and Vimentin were involved in tumorigenesis and the pathologic outcomes of TSCC. We also determined their impact on the survival of TSCC patients with stratification according to various pathologic factors that had previously not been used for such analyses. Most importantly, after evaluating the interactions of E-cadherin, N-cadherin, and pathologic outcomes with survival by Cox regression model, we identified Vimentin as a potential prognostic factor for DSS.

Snail promotes EMT and results in progression and cancer motility in oral SCC [17]. In this study, we found that higher Snail expression was significantly associated with not only tumor development (p<0.001; Table 1) but also advanced stage (p = 0.037, Table 2) of TSCC. In contrast to other two studies on TSCC patients [11,18], the expression of Snail in our study was not correlated with poor cell differentiation [11,18], large tumor size [11], lymph node metastasis [11], or greater invasion depth of the tumor [11,18]. The reason for different clinicopathologic significance may be that the regulation of Snail is complex and modulated by various signals at the transcriptional and post-translational levels in different TSCC populations [19]. One such factor is betel quid chewing, which is an important risk factor for TSCC in Taiwan but not in most Western countries. Moreover, Snail expression was not associated with survival in TSCC (Table 3), thus excluding the possibility of Snail as a potent prognostic factor in TSCC.

Twist is essential for tumor invasion in TSCC cells [13]. However, our data showed that there was no significant difference in Twist expression between TSCC and corresponding TAN tissues (Table 1). Additionally, Twist expression was not correlated with any pathologic outcomes (Table 2) including DSS and DFS in TSCC patients in this study (Table 3). The above results thus suggest that the transcription repression of E-cadherin by Snail but not Twist may promote cell proliferation [20] during tumorigenesis and exclude Twist as a key repressor for tumor metastasis [11,21] in TSCC.

Loss of E-cadherin expression regulated by Snail and Twist promotes the reduction of cell-cell adhesion [22] and results in poor prognosis in OSCC [23]. We found that E-cadherin expression is reduced together with a clearly increase in Snail expression and slight increase in Twist expression in TAN tissues compared to that in tumors (p<0.001; Table 1), indicating the influence of E-cadherin loss in the tumorigenesis of TSCC [24]. In a small cohort of TSCC patients, a previous study indicated that low E-cadherin expression was associated with poor tumor differentiation and higher invasion in TSCC patients [25]. Interestingly, low E-cadherin expression was correlated not only with poor cell differentiation (p<0.001; Table 2) and lymph node metastasis (p = 0.007; Table 2) but also with late pathological stage (p = 0.013; Table 2) and shorter DSS (p = 0.033; Table 3) in our medium-sized cohort of TSCC patients. Additionally, loss of E-cadherin has been shown to be significantly associated with delayed neck metastasis in stage I/II TSCC [21]. Regarding the molecular mechanism of E-cadherin, it has been reported that E-cadherin repression mediated by enhancer of zeste homolog 2 (EZH2) promotes the metastatic phenotype of TSCC cells, and TSCC patients with high expression of EZH2 and low expression of E-cadherin have lower overall survival [26]. Additional analysis is needed to examine the co-expression of EZH2 and E-cadherin in lymph node metastasis in our TSCC patients. In this study, low E-cadherin expression was significantly correlated with shorter DSS in young TSCC patients (≤50 yrs; p = 0.007; S2 Table); this correlation was also observed in gastric cancer patients [27]. The real reason for the impact of E-cadherin on the survival for young patients needs to be further investigated in the near future. Moreover, low E-cadherin expression was significantly correlated with shorter DSS in patients with moderate and poor cell differentiation (p = 0.038; S2 Table), indicating the role of low E-cadherin in poor DSS in TSCC patients with more aggressive phenotypes in terms of cell differentiation. Furthermore, low E-cadherin expression was significantly correlated with shorter DSS in patients without post-operative RT (p = 0.014; S2 Table), which suggests that E-cadherin could be a prognostic marker for certain groups of TSCC patients. However, low E-cadherin expression was not correlated with shorter DFS in patients even after stratification by various clinicopathologic factors (S3 Table).

Usually, high N-cadherin expression is observed in oral tumor tissues [28]. However, our data showed that the level of N-cadherin expression in TAN tissues was higher than that in the tumor (p<0001; Table 1), indicating that N-cadherin may mediate a less stable and more dynamic form of cell-cell adhesion, which may allow both attachment and detachment of individual cells from the primary tumor and selective association with tissues such as the stroma or endothelium in TAN tissues [29]. The other possible reason may be that the higher N-cadherin expression in TAN may influence the gene expression pattern of residual TSCC cells, which may allow them to survive or migrate [30]. In addition, higher N-cadherin expression is characterized by a malignant phenotype associated with worse clinical outcome, increased invasion/metastasis and reduced overall survival in oral cancer patients [12,28]. Co-expression of N-cadherin and β-catenin is essential in TSCC for the down-regulation of the cell cycle inhibitor p21 and up-regulation of MMP-2 and MMP-9, which are considered potential indicators of invasion and metastasis as well as poor survival in TSCC patients [12,31]. Likewise, our results showed that the increased N-cadherin expression was significantly related to poor cell differentiation (p<0.001; Table 2), late pathological stage (p = 0.023; Table 2), and lymph node metastasis (p = 0.007; Table 2), as well as shorter DSS (p = 0.034; Table 3) and DFS (p = 0.022; Table 3) in TSCC.

Vimentin is a predictive biomarker for tumor growth and metastasis, although its significance is limited in TSCC prognosis [32,33]. Our results also showed that Vimentin expression was involved in tumorigenesis (p<0.001; Table 1) and associated with poor cell differentiation (p = 0.006; Table 2) and lymph node metastasis (p = 0.039; Table 2) in TSCC. Surprisingly, Vimentin expression was significantly associated with poor DSS (p<0.001; Table 3) and DFS (p = 0.045; Table 3), and its impact on DSS is appeared to be more important than that of Snail, Twist, E-cadherin and N-cadherin (p = 0.002; Table 4). Three reasons may explain the potential prognostic significance of Vimentin in our study: (1) pathologic outcomes can confound or decrease the impact of Vimentin on DSS; we used the multivariate Cox regression model to adjust the impact of pathologic outcomes on survival; (2) expression levels of E-cadherin, N-cadherin, and Vimentin were quantified simultaneously, and their interaction were also incorporated into the Cox regression model in this study; (3) the sample size of TSCC patients in our study provided adequate statistical power compared to that of studies from other groups, which could have resulted in the identification of prognostic significance of Vimentin in TSCC. Similar to low E-cadherin expression, high Vimentin expression was also significantly correlated with shorter DSS (p = 0.001; S4 Table) in patients with moderate and poor cell differentiation, suggesting that the combination of low E-cadherin expression and high Vimentin expression might be a potential prognostic marker for patients with more differentiated cancer phenotype. However, high Vimentin expression was significantly correlated with shorter DSS in patients with smaller tumors (p = 0.001; S4 Table) and was significantly correlated with shorter DFS in patients with early pathological stage (p = 0.016; S5 Table) and without lymph node metastasis (p = 0.017; S5 Table), implying that Vimentin could be an early prognostic factor in TSCC patients.

Most importantly, Vimentin might be used as a marker to evaluate the prognosis of TSCC patients and help in clinical decision making with respect to the selection of appropriate therapies for individual TSCC patients in the future. More studies are necessary in the future to (a) assess the tumorigenic pathways of Vimentin through its interaction with 14-3-3 and Akt-phosphorylated Beclin 1, which could contribute to control tumorigenesis [34]; (b) understand the regulation of Vimentin through hypoxia-inducible factor-1 and its role in the extracellular space or cell surface during EMT, which could contribute to the invasiveness of cancer cells [35]; (c) understand how Vimentin assembly and expression are regulated, which would help to elucidate the effect of Vimentin expression on metastasis [10]. Furthermore, Vimentin-specific inhibitors as well as antibodies, peptides, aptamers or siRNA directed against Vimentin in combination with other anticancer agents could be used for the treatment of TSCC patients, and more studies are required to further evaluate the therapeutic efficacy of these approached in the clinic.

One possible limitation of our study is that the external validity of our findings is limited to patients in South Asia who are exposed to betel quid chewing, which is not a major risk factor for TSCC among Caucasians. Thus, we further validated these EMT-related proteins in TSCC patients using another independent cohort from The Cancer Genome Atlas (TCGA) database. Consistent with our findings regarding the association of expression levels of the five EMT-related biomarkers with “tumorigenesis”, the expression of Snail (p = 0.009, S6 Table) and Twist (p = 0.001; S6 Table) were significantly higher in tumor tissues than in normal tissues, indicating that the high expression levels of Snail and Twist were reliable biomarkers for tumorigenesis. However, E-cadherin expression (p = 0.621) and Vimentin expression (p = 0.132) were not significantly reduced and elevated, respectively, in tumor tissues compared with the normal tissues. Additionally, N-cadherin expression (p = 0.621) was slightly higher in tumors than in normal tissues; however, no significant difference was found. However, the normal (n = 12) and TSCC tissues (n = 127) were not paired, and few normal tissues are present in the TCGA database; both these factors may help explain the discordant findings between our cohort and the TCGA data. Consistent with our findings regarding the association of expression levels of the five EMT-related biomarkers with “demographic and pathological outcomes”, low E-cadherin expression was only significantly associated with poor cell differentiation (p = 0.001; S7 Table). Moreover, high Vimentin expression was only significantly correlated with lymph node metastasis (p = 0.031; S7 Table). However, the expression levels of Snail and N-cadherin were not associated with any pathological outcomes. For “survival” analysis, the expression levels of all proteins were dichotomized into low and high expression with the same cutoff value as we used in this study. However, the expression levels of these five EMT-related biomarkers were not related to overall survival and disease-free survival (DFS) (S8 Table) since the sample size of patients with DFS was too small (n = 88) compared to the size of our cohort. The possible reasons for the lack of consistency between our study and the results from the TCGA database could be that (a) the five EMT-related proteins in TSCC might be differentially regulated at the mRNA and protein levels[8]; and (b) the regulation of the five EMT-related proteins might be variable due to different environmental exposures and genetic alterations between Asian and Western TSCC patients.

In conclusion, Snail, E-cadherin, N-cadherin, and Vimentin were associated with tumorigenesis and pathologic outcomes in TSCC. Notably, Vimentin may be a potential prognostic factor among the five-EMT related proteins.

## Supporting information

S1 Table The raw clinical data along with expression scores of five EMT-related proteins. (XLS)

S2 Table Impact of E-cadherin expression levels on disease-specific survival by the different clinicopathologic outcomes with TSCC. (DOC)

S3 Table Impact of E-cadherin expression levels on disease-free survival by the different demographic and clinicopathologic factors with TSCC. (DOC)

S4 Table Impact of Vimentin expression levels on disease-specific survival by the different clinicopathologic outcomes with TSCC. (DOC)

S5 Table Impact of Vimentin expression levels on disease-free survival by the different clinicopathologic outcomes with TSCC. (DOC)

S6 Table The comparison of Snail, Twist, E-cadherin, N-cadherin, and Vimentin expression between TSCC and corresponding tumor adjacent normal tissues from TCGA database. (DOC)

S7 Table The correlation of the expression of Snail, Twist, E-cadherin, N-cadherin, and Vimentin to pathological outcomes in TSCC patients from TCGA database. (DOC)

S8 Table The correlation of the expression of Snail, Twist, E-cadherin, N-cadherin, and Vimentin to disease-specific survival and disease-free survival in TSCC patients from TCGA database. (DOC)
