# Supplementary material for: Vimentin is a potential prognostic factor for tongue squamous cell carcinoma among five epithelial–mesenchymal transition-related proteins
Source: PLoS One. 2017 Jun 1;12(6):e0178581. doi: 10.1371/journal.pone.0178581 (PMC5453552; doi:10.1371/journal.pone.0178581)
Supplement: S2 Table — (DOC) [file pone.0178581.s002.doc]

| **S2 Table.** Impact of E-cadherin expression levels on disease-specific survival by the different clinicopathologic outcomes with TSCC. | | | | | | | |
| --- | --- | --- | --- | --- | --- | --- | --- |
| Variable | E-cadherin | No. (%) | CHR (95% CI) | *p value** | AHR (95% CI) | *p value*^†^ | *p* for interaction |
|  |  |  |  |  |  |  |  |
|  |  |  |  |  |  |  |  |
| Sex |  |  |  |  |  |  |  |
| Female | Low | 13 (43.3) | 1.00 |  | 1.00 |  | 0.958 |
|  | High | 17 (56.7) | 0.49 (0.13-1.85) | 0.293 | 0.66 (0.16-2.74) | 0.564^a^ |  |
|  |  |  |  |  |  |  |  |
| Male | Low | 82 (37.6) | 1.00 |  | 1.00 |  |  |
|  | High | 136 (62.4) | 0.56 (0.38-0.83) | **0.004** | 0.66 (0.44-0.98) | **0.037^a^** |  |
| Age, yrs |  |  |  |  |  |  |  |
| ≦50 | Low | 53 (41.4) | 1.00 |  | 1.00 |  | 0.092 |
|  | High | 75 (58.6) | 0.44 (0.27-0.73) | **0.001** | 0.50 (0.30-0.83) | **0.007^a^** |  |
|  |  |  |  |  |  |  |  |
| ＞50 | Low | 42 (35.0) | 1.00 |  | 1.00 |  |  |
|  | High | 78 (65.0) | 0.82 (0.45-1.47) | 0.501 | 0.96 (0.52-1.75) | 0.888**^a^** |  |
| Cell differentiation |  |  |  |  |  |  |  |
| Well | Low | 5 (19.2) | 1.00 |  | 1.00 |  | 0.897 |
|  | High | 21 (80.8) | 1.33 (0.14-12.26) | 0.803 | 1.30 (0.13-12.68) | 0.823^b^ |  |
|  |  |  |  |  |  |  |  |
| Moderate, poor | Low | 90 (40.5) | 1.00 |  | 1.00 |  |  |
|  | High | 132 (59.5) | 0.59 (0.40-0.87) | **0.007** | 0.66 (0.45-0.98) | **0.038^b^** |  |
| AJCC pathological stage |  |  |  |  |  |  |  |
| I, II | Low | 56 (33.3) | 1.00 |  | 1.00 |  | 0.244 |
|  | High | 112 (66.7) | 0.81 (0.47-1.39) | 0.437 | 0.83 (0.48-1.42) | 0.486^c^ |  |
|  |  |  |  |  |  |  |  |
| III, IV | Low | 39 (48.8) | 1.00 |  | 1.00 |  |  |
|  | High | 41 (51.2) | 0.48 (0.28-0.83) | **0.009** | 0.57 (0.33-1.00) | **0.048^c^** |  |
| T classification |  |  |  |  |  |  |  |
| T1, T2 | Low | 73 (37.4) | 1.00 |  | 1.00 |  | 0.320 |
|  | High | 122 (62.6) | 0.61 (0.39-0.95) | **0.030** | 0.74 (0.46-1.16) | 0.189^d^ |  |
|  |  |  |  |  |  |  |  |
| T3, T4 | Low | 22 (41.5) | 1.00 |  | 1.00 |  |  |
|  | High | 31 (58.5) | 0.50 (0.25-0.99) | **0.046** | 0.57 (0.28-1.17) | 0.123^d^ |  |
| N classification |  |  |  |  |  |  |  |
| N0 | Low | 68 (34.7) | 1.00 |  | 1.00 |  | 0.161 |
|  | High | 128 (65.3) | 0.70 (0.44-1.12) | 0.136 | 0.76 (0.48-1.23) | 0.264^e^ |  |
|  |  |  |  |  |  |  |  |
| N1, N2 | Low | 27 (51.9) | 1.00 |  | 1.00 |  |  |
|  | High | 25 (48.1) | 0.48 (0.25-0.93) | **0.030** | 0.50 (0.25-1.02) | 0.057^e^ |  |
| Postoperative RT |  |  |  |  |  |  |  |
| No | Low | 66 (36.5) | 1.00 |  | 1.00 |  | 0.189 |
|  | High | 115 (63.5) | 0.54 (0.33-0.86) | **0.010** | 0.55 (0.34-0.89) | **0.014^a^** |  |
|  |  |  |  |  |  |  |  |
| Yes | Low | 29 (43.3) | 1.00 |  | 1.00 |  |  |
|  | High | 38 (56.7) | 0.69 (0.37-1.27) | 0.229 | 0.81 (0.43-1.54) | 0.521^a^ |  |
| *Abbreviations: CHR, crude hazard ratio; CI, confidence interval; AHR, adjusted hazard ratio; AJCC, American Joint Committee on Cancer; RT, radiotherapy.*  **p values were estimated by Cox’s regression.*  ^†^*p values were estimated by multivariate Cox’s regression.*  *^a^Adjusted for cell differentiation (moderate+poor vs. well) and AJCC pathological stage (stage III+ IV vs. stage I+II).*  *^b^Adjusted for AJCC pathological stage (stage III+ IV vs. stage I+II).*  *^c^Adjusted for cell differentiation (moderate+poor vs. well).*  *^d^Adjusted for cell differentiation (moderate+poor vs. well) and N classification (N1, N2 vs. N0).*  *^e^Adjusted for cell differentiation (moderate+poor vs. well) and T classification (T3, T4 vs. T1, T2).* | | | | | | | |
