# Supplementary material for: Vimentin is a potential prognostic factor for tongue squamous cell carcinoma among five epithelial–mesenchymal transition-related proteins
Source: PLoS One. 2017 Jun 1;12(6):e0178581. doi: 10.1371/journal.pone.0178581 (PMC5453552; doi:10.1371/journal.pone.0178581)
Supplement: S3 Table — (DOC) [file pone.0178581.s003.doc]

| **S3 Table.** Impact of E-cadherin expression levels on disease-free survival by the different demographic and clinicopathologic factors with TSCC. | | | | | | | |
| --- | --- | --- | --- | --- | --- | --- | --- |
| Variable | E-cadherin | No. (%) | CHR (95% CI) | *p value** | AHR (95% CI) | *p value*^†^ | *p* for interaction |
|  |  |  |  |  |  |  |  |
|  |  |  |  |  |  |  |  |
| Sex |  |  |  |  |  |  |  |
| Female | Low | 13 (43.3) | 1.00 |  | 1.00 |  | 0.052 |
|  | High | 17 (56.7) | 0.10 (0.01-0.81) | **0.031** | 0.14 (0.02-1.21) | 0.073^a^ |  |
|  |  |  |  |  |  |  |  |
| Male | Low | 82 (37.6) | 1.00 |  | 1.00 |  |  |
|  | High | 136 (62.4) | 0.81 (0.53-1.23) | 0.326 | 0.85 (0.56-1.30) | 0.459^a^ |  |
| Age, yrs |  |  |  |  |  |  |  |
| ≦50 | Low | 53 (41.4) | 1.00 |  | 1.00 |  | 0.803 |
|  | High | 75 (58.6) | 0.76 (0.43-1.34) | 0.344 | 0.79 (0.44-1.40) | 0.414^a^ |  |
|  |  |  |  |  |  |  |  |
| ＞50 | Low | 42 (35.0) | 1.00 |  | 1.00 |  |  |
|  | High | 78 (65.0) | 0.67 (0.38-1.17) | 0.159 | 0.74 (0.42-1.30) | 0.289**^a^** |  |
| Cell differentiation |  |  |  |  |  |  |  |
| Well | Low | 5 (19.2) | 1.00 |  | 1.00 |  | Incalculable |
|  | High | 21 (80.8) | Incalculable |  | Incalculable |  |  |
|  |  |  |  |  |  |  |  |
| Moderate, poor | Low | 90 (40.5) | 1.00 |  | 1.00 |  |  |
|  | High | 132 (59.5) | 0.72 (0.48-1.09) | 0.120 | 0.74 (0.49-1.12) | 0.150^b^ |  |
| AJCC pathological stage |  |  |  |  |  |  |  |
| I, II | Low | 56 (33.3) | 1.00 |  | 1.00 |  | 0.767 |
|  | High | 112 (66.7) | 0.78 (0.48-1.29) | 0.335 | 0.80 (0.48-1.31) | 0.371^c^ |  |
|  |  |  |  |  |  |  |  |
| III, IV | Low | 39 (48.8) | 1.00 |  | 1.00 |  |  |
|  | High | 41 (51.2) | 0.63 (0.31-1.26) | 0.189 | 0.77 (0.38-1.55) | 0.464^c^ |  |
| T classification |  |  |  |  |  |  |  |
| T1, T2 | Low | 73 (37.4) | 1.00 |  | 1.00 |  | 0.998 |
|  | High | 122 (62.6) | 0.73 (0.46-1.14) | 0.168 | 0.74 (0.47-1.18) | 0.207^d^ |  |
|  |  |  |  |  |  |  |  |
| T3, T4 | Low | 22 (41.5) | 1.00 |  | 1.00 |  |  |
|  | High | 31 (58.5) | 0.68 (0.29-1.60) | 0.371 | 0.85 (0.36-2.02) | 0.719^d^ |  |
| N classification |  |  |  |  |  |  |  |
| N0 | Low | 68 (34.7) | 1.00 |  | 1.00 |  | 0.521 |
|  | High | 128 (65.3) | 0.80 (0.50-1.27) | 0.334 | 0.82 (0.51-1.31) | 0.404^e^ |  |
|  |  |  |  |  |  |  |  |
| N1, N2 | Low | 27 (51.9) | 1.00 |  | 1.00 |  |  |
|  | High | 25 (48.1) | 0.59 (0.26-1.37) | 0.221 | 0.63 (0.27-1.48) | 0.286^e^ |  |
| Postoperative RT |  |  |  |  |  |  |  |
| No | Low | 66 (36.5) | 1.00 |  | 1.00 |  | 0.728 |
|  | High | 115 (63.5) | 0.70 (0.44-1.12) | 0.137 | 0.75 (0.46-1.20) | 0.223^a^ |  |
|  |  |  |  |  |  |  |  |
| Yes | Low | 29 (43.3) | 1.00 |  | 1.00 |  |  |
|  | High | 38 (56.7) | 0.76 (0.36-1.61) | 0.472 | 0.60 (0.27-1.36) | 0.222^a^ |  |
| *Abbreviations: CHR, crude hazard ratio; CI, confidence interval; AHR, adjusted hazard ratio; AJCC, American Joint Committee on Cancer; RT, radiotherapy.*  **p values were estimated by Cox’s regression.*  ^†^*p values were estimated by multivariate Cox’s regression.*  *^a^Adjusted for cell differentiation (moderate+poor vs. well) and AJCC pathological stage (stage III+ IV vs. stage I+II).*  *^b^Adjusted for AJCC pathological stage (stage III+ IV vs. stage I+II).*  *^c^Adjusted for cell differentiation (moderate+poor vs. well).*  *^d^Adjusted for cell differentiation (moderate+poor vs. well) and N classification (N1, N2 vs. N0).*  *^e^Adjusted for cell differentiation (moderate+poor vs. well) and T classification (T3, T4 vs. T1, T2).* | | | | | | | |
