# Supplementary material for: Vimentin is a potential prognostic factor for tongue squamous cell carcinoma among five epithelial–mesenchymal transition-related proteins
Source: PLoS One. 2017 Jun 1;12(6):e0178581. doi: 10.1371/journal.pone.0178581 (PMC5453552; doi:10.1371/journal.pone.0178581)
Supplement: S4 Table — (DOC) [file pone.0178581.s004.doc]

| **S4 Table .** Impact of Vimentin expression levels on disease-specific survival by the different clinicopathologic outcomes with TSCC. | | | | | | | |
| --- | --- | --- | --- | --- | --- | --- | --- |
| Variable | Vimentin | No. (%) | CHR (95% CI) | *p value** | AHR (95% CI) | *p value*^†^ | *p* for interaction |
|  |  |  |  |  |  |  |  |
|  |  |  |  |  |  |  |  |
| Sex |  |  |  |  |  |  |  |
| Female | Low | 26 (86.7) | 1.00 |  | 1.00 |  | 0.456 |
|  | High | 4 (13.3) | 2.05 (0.42-9.87) | 0.373 | 0.97 (0.16-6.01) | 0.970^a^ |  |
|  |  |  |  |  |  |  |  |
| Male | Low | 154 (70.6) | 1.00 |  | 1.00 |  |  |
|  | High | 64 (29.4) | 2.04 (1.36-3.06) | **0.001** | 2.09 (1.39-3.15) | **<0.001^a^** |  |
| Age, yrs |  |  |  |  |  |  |  |
| ≦50 | Low | 93 (72.7) | 1.00 |  | 1.00 |  | 0.209 |
|  | High | 35 (27.3) | 2.96 (1.77-4.94) | **<0.001** | 2.53 (1.51-4.24) | **<0.002^a^** |  |
|  |  |  |  |  |  |  |  |
| ＞50 | Low | 87 (72.5) | 1.00 |  | 1.00 |  |  |
|  | High | 33 (27.5) | 1.36 (0.73-2.52) | 0.328 | 1.58 (0.84-2.98) | 0.158^a^ |  |
| Cell differentiation |  |  |  |  |  |  |  |
| Well | Low | 23 (88.5) | 1.00 |  | 1.00 |  | 0.451 |
|  | High | 3 (11.5) | 2.90 (0.30-28.06) | 0.357 | 3.29 (0.30-36.49) | 0.332^b^ |  |
|  |  |  |  |  |  |  |  |
| Moderate, poor | Low | 157 (70.7) | 1.00 |  | 1.00 |  |  |
|  | High | 65 (29.3) | 1.88 (1.27-2.79) | **0.002** | 1.99 (1.34-2.97) | **0.001^b^** |  |
| AJCC pathological stage |  |  |  |  |  |  |  |
| I, II | Low | 124 (73.8) | 1.00 |  | 1.00 |  | 0.991 |
|  | High | 44 (26.2) | 2.24 (1.30-3.85) | **0.004** | 2.12 (1.23-3.65) | **0.007^c^** |  |
|  |  |  |  |  |  |  |  |
| III, IV | Low | 56 (70.0) | 1.00 |  | 1.00 |  |  |
|  | High | 24 (30.0) | 2.14 (1.20-3.81) | **0.010** | 1.90 (1.07-3.38) | **0.030^c^** |  |
| T classification |  |  |  |  |  |  |  |
| T1, T2 | Low | 139 (71.3) | 1.00 |  | 1.00 |  | 0.668 |
|  | High | 56 (28.7) | 2.42 (1.53-3.82) | **<0.001** | 2.12 (1.34-3.37) | **0.001^d^** |  |
|  |  |  |  |  |  |  |  |
| T3, T4 | Low | 41 (77.4) | 1.00 |  | 1.00 |  |  |
|  | High | 12 (22.6) | 1.88 (0.82-4.30) | 0.134 | 1.57 (0.68-3.59) | 0.291^d^ |  |
| N classification |  |  |  |  |  |  |  |
| N0 | Low | 147 (75.0) | 1.00 |  | 1.00 |  | 0.579 |
|  | High | 49 (25.0) | 1.96 (1.20-3.19) | **0.007** | 1.97 (1.20-3.23) | **0.008^e^** |  |
|  |  |  |  |  |  |  |  |
| N1, N2 | Low | 33 (63.5) | 1.00 |  | 1.00 |  |  |
|  | High | 19 (36.5) | 2.04 (1.06-3.93) | **0.034** | 1.92 (0.97-3.78) | 0.060^e^ |  |
| Postoperative RT |  |  |  |  |  |  |  |
| No | Low | 132 (72.9) | 1.00 |  | 1.00 |  | 0.818 |
|  | High | 49 (27.1) | 2.03 (1.24-3.31) | **0.005** | 2.01 (1.22-3.30) | **0.006^a^** |  |
|  |  |  |  |  |  |  |  |
| Yes | Low | 48 (71.6) | 1.00 |  | 1.00 |  |  |
|  | High | 19 (28.4) | 2.37 (1.24-4.54) | **0.009** | 2.21 (1.15-4.26) | **0.017^a^** |  |
| *Abbreviations: CHR, crude hazard ratio; CI, confidence interval; AHR, adjusted hazard ratio; AJCC, American Joint Committee on Cancer; RT, radiotherapy.*  **p values were estimated by Cox’s regression.*  ^†^*p values were estimated by multivariate Cox’s regression.*  *^a^Adjusted for cell differentiation (moderate+poor vs. well) and AJCC pathological stage (stage III+ IV vs. stage I+II).*  *^b^Adjusted for AJCC pathological stage (stage III+ IV vs. stage I+II).*  *^c^Adjusted for cell differentiation (moderate+poor vs. well).*  *^d^Adjusted for cell differentiation (moderate+poor vs. well) and N classification (N1, N2 vs. N0).*  *^e^Adjusted for cell differentiation (moderate+poor vs. well) and T classification (T3, T4 vs. T1, T2).* | | | | | | | |
