# Supplementary material for: Vimentin is a potential prognostic factor for tongue squamous cell carcinoma among five epithelial–mesenchymal transition-related proteins
Source: PLoS One. 2017 Jun 1;12(6):e0178581. doi: 10.1371/journal.pone.0178581 (PMC5453552; doi:10.1371/journal.pone.0178581)
Supplement: S5 Table — (DOC) [file pone.0178581.s005.doc]

| **S5 Table .** Impact of Vimentin expression levels on disease-free survival by the different clinicopathologic outcomes with TSCC. | | | | | | | |
| --- | --- | --- | --- | --- | --- | --- | --- |
| Variable | Vimentin | No. (%) | CHR (95% CI) | *p value** | AHR (95% CI) | *p value*^†^ | *p* for interaction |
|  |  |  |  |  |  |  |  |
|  |  |  |  |  |  |  |  |
| Sex |  |  |  |  |  |  |  |
| Female | Low | 26 (86.7) | 1.00 |  | 1.00 |  | 0.084 |
|  | High | 4 (13.3) | 6.00 (1.34-26.90) | **0.019** | 3.38 (0.55-20.80) | 0.189^a^ |  |
|  |  |  |  |  |  |  |  |
| Male | Low | 154 (70.6) | 1.00 |  | 1.00 |  |  |
|  | High | 64 (29.4) | 1.44 (0.93-2.22) | 0.101 | 1.37 (0.88-2.11) | 0.161^a^ |  |
| Age, yrs |  |  |  |  |  |  |  |
| ≦50 | Low | 93 (72.7) | 1.00 |  | 1.00 |  | 0.800 |
|  | High | 35 (27.3) | 1.57 (0.86-2.87) | 0.140 | 1.49 (0.81-2.75) | 0.202^a^ |  |
|  |  |  |  |  |  |  |  |
| ＞50 | Low | 87 (72.5) | 1.00 |  | 1.00 |  |  |
|  | High | 33 (27.5) | 1.70 (0.95-3.03) | 0.073 | 1.69 (0.94-3.04) | 0.079^a^ |  |
| Cell differentiation |  |  |  |  |  |  |  |
| Well | Low | 23 (88.5) | 1.00 |  | 1.00 |  | 0.617 |
|  | High | 3 (11.5) | 2.38 (0.25-22.93) | 0.452 | 1.72 (0.18-16.51) | 0.640^b^ |  |
|  |  |  |  |  |  |  |  |
| Moderate, poor | Low | 157 (70.7) | 1.00 |  | 1.00 |  |  |
|  | High | 65 (29.3) | 1.53 (1.00-2.33) | 0.052 | 1.51 (0.99-2.31) | 0.059^b^ |  |
| AJCC pathological stage |  |  |  |  |  |  |  |
| I, II | Low | 124 (73.8) | 1.00 |  | 1.00 |  | 0.245 |
|  | High | 44 (26.2) | 1.93 (1.17-3.20) | **0.010** | 1.86 (1.13-3.08) | **0.016^c^** |  |
|  |  |  |  |  |  |  |  |
| III, IV | Low | 56 (70.0) | 1.00 |  | 1.00 |  |  |
|  | High | 24 (30.0) | 1.16 (0.55-2.46) | 0.700 | 0.99 (0.47-2.11) | 0.986^c^ |  |
| T classification |  |  |  |  |  |  |  |
| T1, T2 | Low | 139 (71.3) | 1.00 |  | 1.00 |  | 0.965 |
|  | High | 56 (28.7) | 1.66 (1.04-2.65) | **0.032** | 1.60 (1.00-2.57) | 0.051^d^ |  |
|  |  |  |  |  |  |  |  |
| T3, T4 | Low | 41 (77.4) | 1.00 |  | 1.00 |  |  |
|  | High | 12 (22.6) | 1.66 (0.64-4.32) | 0.298 | 1.28 (0.49-3.34) | 0.616^d^ |  |
| N classification |  |  |  |  |  |  |  |
| N0 | Low | 147 (75.0) | 1.00 |  | 1.00 |  | 0.207 |
|  | High | 49 (25.0) | 1.89 (1.17-3.04) | **0.009** | 1.79 (1.11-2.88) | **0.017^e^** |  |
|  |  |  |  |  |  |  |  |
| N1, N2 | Low | 33 (63.5) | 1.00 |  | 1.00 |  |  |
|  | High | 19 (36.5) | 0.96 (0.41-2.27) | 0.923 | 0.94 (0.39-2.28) | 0.898^e^ |  |
| Postoperative RT |  |  |  |  |  |  |  |
| No | Low | 132 (72.9) | 1.00 |  | 1.00 |  | 0.590 |
|  | High | 49 (27.1) | 1.54 (0.94-2.52) | 0.087 | 1.37 (0.83-2.24) | 0.216^a^ |  |
|  |  |  |  |  |  |  |  |
| Yes | Low | 48 (71.6) | 1.00 |  | 1.00 |  |  |
|  | High | 19 (28.4) | 1.99 (0.91-4.35) | 0.087 | 2.11 (0.96-4.66) | 0.065^a^ |  |
| *Abbreviations: CHR, crude hazard ratio; CI, confidence interval; AHR, adjusted hazard ratio; AJCC, American Joint Committee on Cancer; RT, radiotherapy.*  **p values were estimated by Cox’s regression.*  ^†^*p values were estimated by multivariate Cox’s regression.*  *^a^Adjusted for cell differentiation (moderate+poor vs. well) and AJCC pathological stage (stage III+ IV vs. stage I+II).*  *^b^Adjusted for AJCC pathological stage (stage III+ IV vs. stage I+II).*  *^c^Adjusted for cell differentiation (moderate+poor vs. well).*  *^d^Adjusted for cell differentiation (moderate+poor vs. well) and N classification (N1, N2 vs. N0).*  *^e^Adjusted for cell differentiation (moderate+poor vs. well) and T classification (T3, T4 vs. T1, T2).* | | | | | | | |
