# Supplementary material for: Vimentin is a potential prognostic factor for tongue squamous cell carcinoma among five epithelial–mesenchymal transition-related proteins
Source: PLoS One. 2017 Jun 1;12(6):e0178581. doi: 10.1371/journal.pone.0178581 (PMC5453552; doi:10.1371/journal.pone.0178581)
Supplement: S6 Table — (DOC) [file pone.0178581.s006.doc]

| **Table S6.** The comparison of Snail, Twist, E-cadherin, N-cadherin, and Vimentin expression between TSCC and corresponding tumor adjacent normal tissues from TCGA database. | | | | | | | |
| --- | --- | --- | --- | --- | --- | --- | --- |
| Variables | Solid Tissue Normal (n=12) | |  | Tumor (n=127) | | Z | *p*-value^＊^ |
|  | Mean±SD | Median |  | Mean±SD | Median |  |  |
| Snail | 5.85±0.68 | 6.10 |  | 6.61±1.05 | 6.59 | -2.610 | **0.009** |
|  |  |  |  |  |  |  |  |
| Twist | 6.18±1.15 | 6.22 |  | 7.50±1.19 | 7.53 | -3.412 | **0.001** |
|  |  |  |  |  |  |  |  |
| E-cadherin | 13.15±0.73 | 13.35 |  | 12.99±1.25 | 13.23 | -0.495 | 0.621 |
|  |  |  |  |  |  |  |  |
| N-cadherin | 5.28±1.24 | 4.84 |  | 5.82±2.28 | 5.69 | -0.907 | 0.364 |
|  |  |  |  |  |  |  |  |
| Vimentin | 13.27±1.02 | 12.93 |  | 13.73±1.15 | 13.70 | -1.507 | 0.132 |
| *Abbreviations: TSCC, tongue squamous cell carcinoma; SD, standard deviation.*  ^＊^*p-values* *were estimated by Mann-Whitney U test.* | | | | | | | |
