# Supplementary material for: Vimentin is a potential prognostic factor for tongue squamous cell carcinoma among five epithelial–mesenchymal transition-related proteins
Source: PLoS One. 2017 Jun 1;12(6):e0178581. doi: 10.1371/journal.pone.0178581 (PMC5453552; doi:10.1371/journal.pone.0178581)
Supplement: S7 Table — (DOC) [file pone.0178581.s007.doc]

| **Table S7.** The correlation of the expression of Snail, Twist, E-cadherin, N-cadherin, and Vimentin to pathological outcomes in TSCC patients from TCGA database. | | | | | | | | | | | | | | | |
| --- | --- | --- | --- | --- | --- | --- | --- | --- | --- | --- | --- | --- | --- | --- | --- |
| Variable | No. (%) | Snail | |  | Twist | |  | E-cadherin | |  | N-cadherin | |  | Vimentin | |
|  |  | Mean±SD  (median) | *p value* |  | Mean±SD (median) | *p value* |  | Mean±SD  (median) | *p value* |  | Mean±SD (median) | *p value* |  | Mean±SD  (median) | *p value* |
| Sex |  |  |  |  |  |  |  |  |  |  |  |  |  |  |  |
| Female | 44 (34.6) | 6.50±0.99 (6.63) | 0.389^＊^ |  | 7.34±0.98 (7.43) | 0.273^＊^ |  | 13.16±0.68 (13.32) | 0.283^＊^ |  | 5.54±2.43 (5.47) | 0.324^＊^ |  | 13.63±1.19 (13.69) | 0.466^＊^ |
| Male | 83 (65.4) | 6.66±1.08 (6.59) |  |  | 7.59±1.28 (7.64) |  |  | 12.91±1.47 (13.15) |  |  | 5.96±2.20 (5.92) |  |  | 13.78±1.13 (13.75) |  |
| Age, y |  |  |  |  |  |  |  |  |  |  |  |  |  |  |  |
| ≦40 | 12 (9.4) | 5.97±0.98 (5.95) | 0.089^†^ |  | 7.54±0.94 (7.70) | 0.331^†^ |  | 13.48±0.42 (13.49) | 0.324^†^ |  | 5.56±2.20 (5.82) | 0.358^†^ |  | 13.54±1.42 (13.68) | 0.622^†^ |
| 41-50 | 22 (17.3) | 6.82±0.94 (6.73) |  |  | 7.88±1.19 (7.98) |  |  | 13.20±0.61 (13.32) |  |  | 6.59±2.52 (6.57) |  |  | 13.81±1.21 (13.79) |  |
| 51-60 | 32 (25.2) | 6.48±1.09 (6.52) |  |  | 7.27±1.16 (7.25) |  |  | 12.78±1.23 (13.17) |  |  | 5.54±1.95 (5.16) |  |  | 13.54±1.09 (13.40) |  |
| ＞60 | 61 (48.0) | 6.72±1.05 (6.64) |  |  | 7.48±1.23 (7.53) |  |  | 12.94±1.50 (13.13) |  |  | 5.73±2.35 (5.58) |  |  | 13.83±1.10 (13.95) |  |
| Cell differentiation | |  |  |  |  |  |  |  |  |  |  |  |  |  |  |
| Well | 16 (12.6) | 6.35±0.70 (6.39) | 0.488^†^ |  | 7.29±0.80 (7.41) | 0.231^†^ |  | 13.42±0.61 (13.55)^a^ | **0.010^†^** |  | 5.30±1.43(5.76) | 0.061^‡^ |  | 13.44±0.98 (13.54) | 0.245^†^ |
| Moderate | 87 (68.5) | 6.61±1.06 (6.59) |  |  | 7.44±1.23 (7.52) |  |  | 13.10±0.92 (13.31)^b^ |  |  | 5.64±2.07 (5.54) |  |  | 13.70±1.14 (13.67) |  |
| Poor | 24 (18.9) | 6.76±1.21 (7.01) |  |  | 7.86±1.19 (7.99) |  |  | 12.33±2.14 (12.72)^ab^ |  |  | 6.81±3.13 (7.35) |  |  | 14.04±1.24 (14.24) |  |
| AJCC pathological stage | |  |  |  |  |  |  |  |  |  |  |  |  |  |  |
| I, II | 39 (30.7) | 6.56±0.97 (6.58) | 0.734^*^ |  | 7.40±1.25 (7.34) | 0.515^＊^ |  | 12.89±1.78 (13.23) | 0.525^＊^ |  | 5.62±2.64 (5.52) | 0.508^§^ |  | 13.70±1.14 (13.85) | 0.873^*^ |
| III, IV | 88 (69.3) | 6.63±1.09 (6.60) |  |  | 7.55±1.16 (7.61) |  |  | 13.04±0.94 (13.22) |  |  | 5.91±2.11 (5.87) |  |  | 13.74±1.16 (13.69) |  |
| T classification | |  |  |  |  |  |  |  |  |  |  |  |  |  |  |
| T1, T2 | 70 (55.1) | 6.58±0.95 (6.58) | 0.775^＊^ |  | 7.52±1.19 (7.57) | 0.837^＊^ |  | 12.94±1.43 (13.20) | 0.586^＊^ |  | 5.85±2.38 (5.76) | 0.874^＊^ |  | 13.86±1.10 (13.89) | 0.156^＊^ |
| T3, T4 | 57 (44.9) | 6.64±1.17 (6.68) |  |  | 7.48±1.20 (7.46) |  |  | 13.06±1.00 (13.27) |  |  | 5.78±2.16 (5.69) |  |  | 13.57±1.19 (13.67) |  |
| N classification | |  |  |  |  |  |  |  |  |  |  |  |  |  |  |
| N0 | 63 (49.6) | 6.53±1.06 (6.59) | 0.434^＊^ |  | 7.42±1.26 (7.42) | 0.416^＊^ |  | 13.03±1.45 (13.27) | 0.718^＊^ |  | 5.58±2.37 (5.64) | 0.241^*^ |  | 13.51±1.20 (13.53) | **0.031^＊^** |
| N1, N2 | 64 (50.4) | 6.68±1.04 (6.60) |  |  | 7.59±1.11 (7.64) |  |  | 12.95±1.04 (13.18) |  |  | 6.05±2.17 (5.93) |  |  | 13.94±1.05 (13.83) |  |
| *Abbreviations: TSCC, tongue squamous cell carcinoma; AJCC, American Joint Committee on Cancer.*  ^＊^*p values were estimated by Student’s t-test.*  ^†^ *p values were estimated by one-way ANOVA test.*  ^‡^*p values were estimated by Kruskal-Wallis one-way ANOVA test.*  ^a^p=0.025; ^b^p=0.027. | | | | | | | | | | | | | | | |
