# Supplementary material for: Vimentin is a potential prognostic factor for tongue squamous cell carcinoma among five epithelial–mesenchymal transition-related proteins
Source: PLoS One. 2017 Jun 1;12(6):e0178581. doi: 10.1371/journal.pone.0178581 (PMC5453552; doi:10.1371/journal.pone.0178581)
Supplement: S8 Table — (DOC) [file pone.0178581.s008.doc]

| **Table S8.** The correlation of the expression of Snail, Twist, E-cadherin, N-cadherin, and Vimentin to disease-specific survival and disease-free survival in TSCC patients from TCGA database. | | | | | | | | |
| --- | --- | --- | --- | --- | --- | --- | --- | --- |
| Variable | | Overall survival (n=125) | | |  | Disease-free survival (n=88) | | |
|  |  | No. (%) | AHR (95% CI) | *p value** |  | No. (%)^†^ | AHR (95% CI) | *p value** |
| Snail expression | Low | 73 (58.4) | 1.00 |  |  | 53 (60.2) | 1.00 |  |
|  | High | 52 (41.6) | 0.86 (0.47-1.59) | 0.634 |  | 35 (39.8) | 0.67 (0.27-1.67) | 0.388 |
| Twist expression | Low | 74 (59.2) | 1.00 |  |  | 55 (62.5) | 1.00 |  |
|  | High | 51 (40.8) | 0.87 (0.47-1.62) | 0.660 |  | 33 (37.5) | 0.45 (0.17-1.25) | 0.125 |
| E-cadherin expression | Low | 48 (38.4) | 1.00 |  |  | 38 (43.2) | 1.00 |  |
|  | High | 77 (61.6) | 0.73 (0.40-1.35) | 0.319 |  | 50 (56.8) | 0.48 (0.20-1.18) | 0.109 |
| N-cadherin expression | Low | 115 (92.0) | 1.00 |  |  | 81 (92.0) | 1.00 |  |
|  | High | 10 (8.0) | 0.88 (0.30-2.57) | 0.809 |  | 7 (8.0) | 0.68 (0.16-2.98) | 0.613 |
| Vimentin | Low | 91 (72.8) | 1.00 |  |  | 65 (73.9) | 1.00 |  |
|  | High | 34 (27.2) | 0.85 (0.41-1.75) | 0.651 |  | 23 (26.1) | 0.60 (0.19-1.68) | 0.301 |
| *Abbreviations: TSCC, tongue squamous cell carcinoma; AHR, adjusted hazard ratio; CI, confidence interval.*  **p-value were adjusted for cell differentiation(moderate+poor vs. well) and AJCC pathological stage (stage III+IV vs stage I+II) by multiple Cox‘s regression.* | | | | | | | | |
